# Performance, priorities, and future of biomedical research publications in Africa: Need for networks between scientists in developed and developing countries.

**Published:** 2008-03-28

**Authors:** Olalekan A Uthman

**Affiliations:** 1 Center for Evidence-Based Global Health, Nigeria

**Keywords:** doctors, health workers, migration, Africa

Scientific publications play an important role in the scientific process providing a key linkage between knowledge production and use [1]. Africa’s health challenges and research needs have been well documented for many years now. Scientific publishing activity worldwide over the past decades shows that most countries in Africa have low levels of publication [1–10]. In the 1990s, the term “the 10/90” gap was coined to express the acute global imbalance whereby developing countries experienced 90 per cent of the world’s major health problems, but received only 10 per cent of its resources for health research [11–13].

The difficulties in research, publication, editorial bias, and information access facing Africa are profound and seem almost intractable [1, 14]. Richard Horton, editor-in-chief of The Lancet in his excellent discussion of this issue has highlighted some of the barriers to information exchange between North and South [14]. Lack of funding, poor laboratory, limited technical support, little available training, few tutors or mentors, no career structure, weak peer networks, diffuse relation between research and academic reward, and research biased to Northern interests are important factors affecting research production from Africa. The low proportions of published articles from authors from Africa have been reported in many research fields [1–10], and may be due to fear of rejection, uncertain about journal options, and no culture of publication to draw on. In addition, serious under-representation of editorial and advisory board members from countries with a low human development index in medical journals has been documented recently [15–17]. This editorial bias may be due to low interest in less-developed countries and reviews fail to take account of local research conditions. Another difficulty facing African researcher is dissemination of findings to other parts of the world. Most of the information published in African journals are largely not included in major databases. Access to technological tools, information access, and other equipment and supplies to ease the work is not always possible. Researchers in Africa are poorly paid [18, 19]. Many have to work in private practice to make ends meet. Many research units in Africa are struggling to cope with a “brain drain” of basic scientist and clinical researchers to developed countries which offer more opportunities and greater political and financial securities [20–23].

There is an urgent need to strengthen local research capacity in Africa, to tackle compelling questions about health and disease vital to enhancing people’s health, lives and livelihoods. More support should be provided by developed countries to Africa, for the advancement of local research efforts. This support should aim to improve the infrastructure of research in Africa. In addition, there is a need for Africa countries to develop and sustain research capacity by attracting, developing and retaining excellent scientists, providing and sustaining the infrastructure and equipment that high-quality research needs; developing strong research institutions with effective leadership, governance and management systems; sustaining a balanced research portfolio in the Africa and partner countries. Researchers from Africa should work effectively through partnerships internationally, nationally; regionally and locally to ensure that research agenda is relevant to Africa’s need and deliverable. There is a need for schemes to promote research as a viable career option and by giving more research awards and supplementing researchers’ salaries. This can be inform of non-bond research grants to PhD students studies in local universities that are have a good links to reputable institutions in the North. Researchers from Africa should join national, regional, and global networks. Developing computerized knowledge management systems to more accurately track research output including grey literature may help eliminate intellectual isolation.

Health research priorities in Africa have often been determined by funders, institutions or individuals, rather than by means of a truly participatory and rational process. There is a need to strengthen the “African Voice” in determining the priorities for future health research. On a final note, I believe like others, that adopting a philosophy of friends-helping-friends and intellectual solidarity will help promotes a commitment to research to equity in health development in Africa.
